# Diffusion Weighted Imaging Evaluated the Early Therapy Effect of Tamoxifen in an MNU-Induced Mammary Cancer Rat Model

**DOI:** 10.1371/journal.pone.0064445

**Published:** 2013-05-21

**Authors:** Guihua Zhai, Clinton J. Grubbs, Cecil R. Stockard, Heidi R. Umphrey, T. Mark Beasley, Hyunki Kim

**Affiliations:** 1 The Department of Radiology, The University of Alabama at Birmingham, Birmingham, Alabama, United States of America; 2 The Department of Surgery, The University of Alabama at Birmingham, Birmingham, Alabama, United States of America; 3 The Department of Biostatistics, The University of Alabama at Birmingham, Birmingham, Alabama, United States of America; 4 Comprehensive Cancer Center, The University of Alabama at Birmingham, Birmingham, Alabama, United States of America; Mie University Graduate School of Medicine, Japan

## Abstract

**Purpose:**

To assess the optimal time point of diffusion-weighted imaging (DWI) for early prognosis of breast cancer following tamoxifen therapy using a methylnitrosourea (MNU)-induced ER-positive breast-cancer model.

**Methods:**

Two groups of Sprague-Dawley rats (n = 15 for group 1; n = 10 for group 2) were used. All animals (50 days old) were intravenously injected with MNU (50 mg/kg body weight) to induce ER-positive mammary tumors. When tumors were approximately 2 cm in diameter, DWI was performed on days 0, 3, and 7, and intratumoral apparent diffusion coefficient (ADC) values were measured. Therapy started on day 0 with tamoxifen (10 mg/kg diet) and continued for 4 weeks for group 1, but only 1 week for group 2, while tumor volume was measured by caliper twice weekly. All animals of group 2 were euthanized on day 7 after imaging, and Ki-67, TUNEL, ERα, and ERβ staining were performed on tumor tissue.

**Results:**

DW images of MNU-induced mammary tumors were successfully obtained with minimal motion artifact. For group 1, ADC change for 3 days after therapy initiation (ADC_3D_) was significantly correlated with tumor-volume change until day 11, but the significant correlation between ADC change for 7 days (ADC_7D_) and the tumor-volume change was observed until day 18. Similarly, for group 2, either ADC_7D_ or ADC_3D_ was significantly correlated with the tumor-volume change, but the higher significance was observed for ADC_7D_. Furthermore, ADC_7D_ was significantly correlated with apoptotic (TUNEL stained), proliferative (Ki-67 stained), and ERβ-positive cell densities, but ADC_3D_ was not significantly correlated with any of those.

**Conclusions:**

ADC_7D_ might be a more reliable surrogate imaging biomarker than ADC_3D_ to assess effectiveness of tamoxifen therapy for ER-positive breast cancer, which may enable personalized treatment. The significant correlation between ADC_7D_ and ERβ-positive cell density suggests that ERβ may play an important role as a therapeutic indicator of tamoxifen.

## Introduction

Estrogen stimulates cell growth via binding to the estrogen receptor (ER), and approximately 75% of breast cancers in the United States are ER positive. Thus, anti-ER drugs like tamoxifen, toremifene, and fulvestrant have been used for treatment of both early and advanced ER positive breast cancers [1], [2], [3]. Tamoxifen is the standard anti-ER therapeutic agent for breast cancer (approved by FDA) in the neoadjuvant as well as adjuvant settings [4], [5]; tamoxifen reduces tumor size in responding patients to facilitate conservation surgery without affecting survival, and it decreases the recurrence rate up to 50% regardless of menopausal status. However, tamoxifen has presented a wide range of sensitivity in individuals [6]. Because the characteristics of breast cancer vary among patients, it would be ideal to tailor the therapeutic strategy to each patient.

Individualized optimal treatment, called personalized medicine, can be guided by molecular biomarkers obtained from biopsies, or by the use of imaging biomarkers. Although minimally invasive biopsy techniques are available [7], they can be associated with pain and stress to patients. Therefore, non-invasive imaging is a better approach to address response to therapy. Diffusion weighted imaging (DWI) is a physiologic MRI (magnetic resonance imaging) modality, which enables quantification of the amplitude of water mobility due to thermodynamic effect as apparent diffusion coefficient (ADC) value [8]. During apoptosis or necrosis induced by effective therapy, water in the extra-cellular space is increased, and this change is detectable prior to visible change of tumor morphology or size. DWI has been validated as a prognostic tool to monitor the breast-cancer response following chemotherapy [9], [10], [11]. However, the decision of therapy effectiveness would be highly dependent upon the imaging time point post therapy initiation, since ADC values change non-linearly over time. For example, the significant increase of ADC value was detected in breast tumor xenografts at 3 days after anti-DR5 (death receptor 5) therapy, but not at day 6 in our previous study, presumably because water molecules diffused away from the tumor region over time [12].

The primary goal of this study was to estimate the optimal DWI time point, evaluating tamoxifen therapy in higher accuracy, using a methylnitrosourea (MNU)-induced ER-positive breast-cancer murine model. Standard cell-line driven xenograft models, routinely used for drug testing, poorly predict for human cancer response. Tumor cells in culture have selective pressure resulting in less differentiated but more homogeneous cells; therefore, the tumors implanted into animals no longer maintain original characteristics [13]. In contrast, the MNU model of mammary carcinoma has higher resemblance to human breast cancer in the aspects of the morphology, origin, and preinvasive stages; this model has been extensively used for testing chemotherapeutic or chemopreventive drugs [14], [15].

The secondary goal was to explore the potential of ERα and ERβ as prognostic biomarkers for tamoxifen therapy. Estrogen receptors are generally classified into two different kinds such as ERα and ERβ [16], [17], [18]. Tamoxifen has been well known to engage ERα as an antagonist to suppress the estrogen-stimulated cell growth [19], but the role of ERβ is still unclear. In this study, the cellular densities of ERα and ERβ were analyzed with immunohistochemistry, and their correlation with tumor-volume regression and ADC increase were determined.

## Materials and Methods

### Reagents

All reagents were from Fisher (Pittsburg, PA) unless otherwise specified. MNU was purchased from National Cancer Institute (NCI) Chemical Repository (Bethesda, MD), and teklad mash diet was purchased from Harlan Teklad (Madison, WI). Tamoxfien was purchased from Agvar Chemical, Inc (Little Falls, NJ).

### Animal preparation

**Figure 1:**
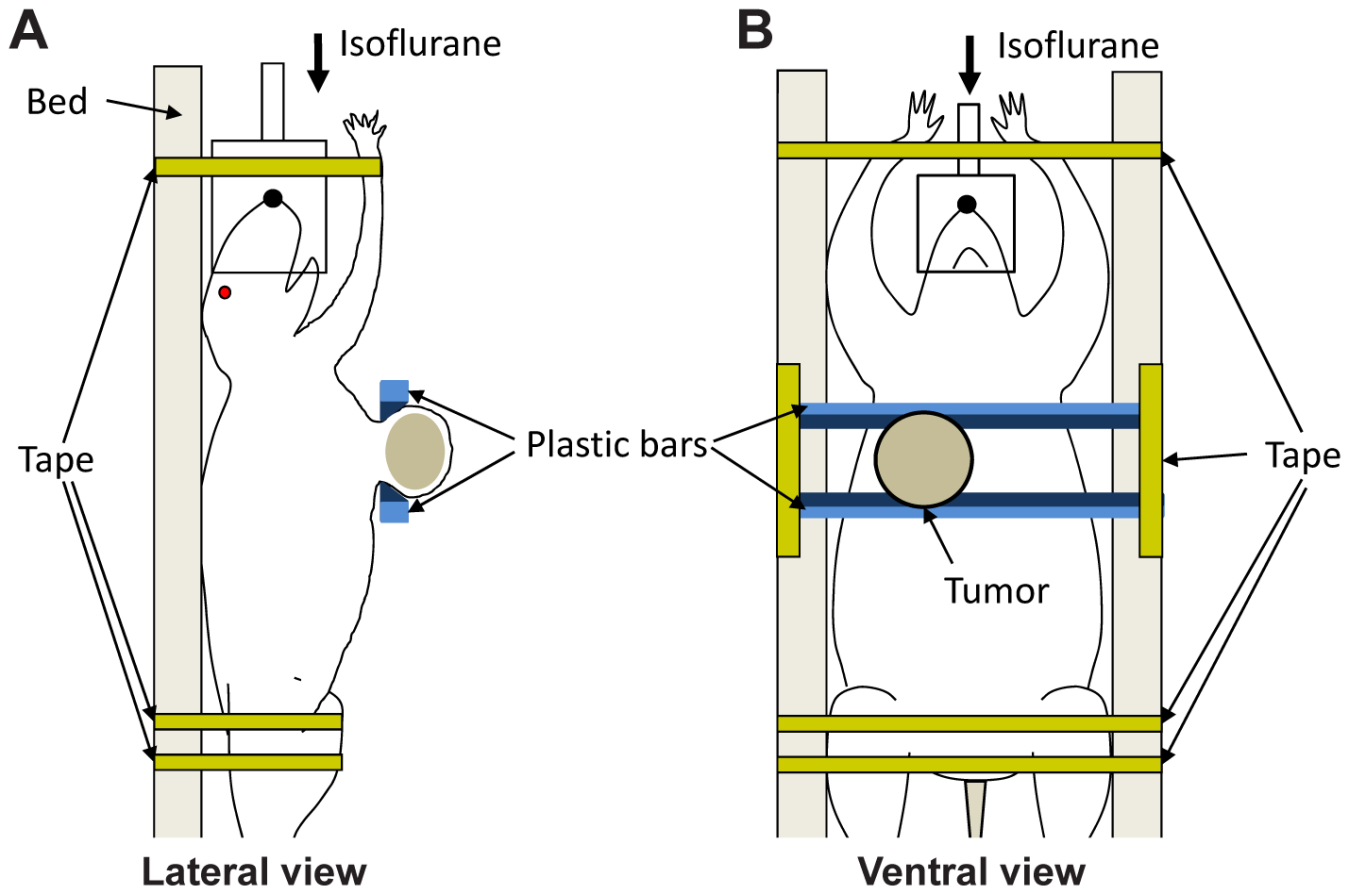
Illustration of animal positioning for DWI. (A) Lateral and (B) ventral views of an animal with a mammary tumor. Two plastic bars were used to lift up the tumor to minimize motion artifact. Wedges were represented with dark blue.

This study strictly followed the recommendations of National Institute of Health (NIH) for the Care and Use of Laboratory Animals. Animal experiments were reviewed and approved by the Committee on the Ethics of Animal Experiments of the University of Alabama at Birmingham (Permit Number: 120609080). All efforts were made for minimizing animal suffering. Two groups of Sprague-Dawley rats (Harlan Sprague Dawley, Inc., Indianapolis, IN; n = 15 for group 1; n = 10 for group 2) were used. When the rats were 50 days old, they were injected with MNU (50 mg/kg body weight) through the left jugular vein to induce mammary tumors. Tumors formed in multiple places, and one tumor of about 2 cm in diameter was selected. T2-weighted MR imaging and DWI were performed for each tumor on day 0 (baseline), and days 3 and 7 after the initiation of tamoxifen therapy. Two plastic bars with wedges were used to lift up the tumor to separate it from respiratory motion, as illustrated in Figure 1. After imaging on day 0, the rats were fed on teklad rodent diet with tamoxifen (10 mg/kg diet); tamoxifen dose was adjusted to induce differential therapeutic efficacy among animals. For group 1, therapy continued for 4 weeks; tumor volume was measured using T2W MR images for the first week but, during the following 3 weeks, it was calculated using the equation, 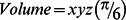, where 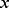, 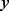, and 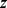 are three orthogonal dimensions measured by a caliper. Measurement was done twice weekly. Animals of group 1 were categorized into three sub-groups based on the time to achieve 50% reduction of tumor volume such as sensitive (less than a week), intermediate (1∼2 weeks), and resistant groups (longer than 2 weeks). For group 2, the rats were sacrificed after imaging on day 7, and the imaged tumors were collected for histological analysis. Animals of group 2 were also categorized into three sub-groups based on tumor volume reduction (%) during one week after therapy initiation such as sensitive (more than 50%), intermediate (10∼50%), and resistant groups (less than 10%), while tumor volume was measured using T2W MR images. All animals were anesthetized using isoflurane gas (1∼2%) during imaging.

### MR imaging

Small-animal imaging was performed on a 9.4T MR imaging system (BioSpec; Bruker BioSpin, Billerica, Mass) with a surface coil (Bruker BioSpin) as a receiver. An MR imaging compatible small-animal respiratory gating device (SA instrument, Stony Brook, NY) was used. Anatomic MR images were acquired with a T2-weighted (T2W) fast spin echo sequence (rapid acquisition with relaxation enhancement) with the following parameters: NEX = 1, TR = 3000 ms, TE = 34 ms, rare factor = 4, FOV = 30×30 mm, and matrix size = 128×128. Continuous 1-mm thick slices were used to cover the entire tumor region. Tumor volume was calculated by summing all voxels inside of the tumor boundary of the anatomical MR images. Diffusion weighted imaging was obtained with a diffusion weighted multi-slice 2-dimensional spin echo sequence. Four *b* values (5, 300, 600, and 1000 s/mm^2^) were applied in the *x* direction with the following parameters: NEX = 1, TR = 3500 ms, TE = 32 ms, diffusion separation time = 16 ms, diffusion gradient duration = 6 ms, FOV = 30×30 mm, and matrix size = 128×128. A total of seven 1-mm thick slices were used to cover the central tumor region, and ADC value was averaged over all those slices. ADC value was calculated by finding the best fitting curve to the equation, 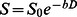, where *S* is the intensity of DW images, *S_0_* is a constant, and *D* is ADC value. The tumor region of interest (ROI) was determined in T2W images; skin and connective tissue were manually excluded based on tumor anatomy, and then global thresholding technique was applied using ImageJ (version 1.45i; NIH, Bethesda, MD), while threshold value was manually determined for each image slice. The ROI obtained from T2W images was also used to determine the tumor region in ADC maps; minimal geometrical distortion was observed even in the high-*b*-value DW images. Tumor volume and ADC quantification was implemented with software developed with Labview 2010, version 10.0.1 (National Instruments Co., Austin, TX). Dr. Zhai implemented all MR image analyses.

### Histological analysis

Ki67 and TUNEL (terminal deoxynucleotidyl transferase mediated dUTP nick end labeling) staining were performed for tumor tissues of group 2 with the same procedure as reported previously [12]. ERα or ERβ staining was performed for the tumor tissues with the following procedure; anti-ERα and anti-ERβ antibodies (Abcam plc, Boston, MA) diluted 1∶200 were placed on the tissue sections and incubated overnight at 4°C. Secondary antibody, HRP (horseradish peroxidase) conjugated goat anti-rabbit antibody (Jackson Immuno Research, West Grove, PA), was diluted 1∶200 in azide free tris/triton X-100 buffer and placed on the tissue sections. Thereafter, the tissue sections were incubated at room temperature for 40 minutes, and DAB (3,3′ diaminobenzidine) chromagen (Scy Tek Laboratories, Logan, UT) was applied for 7 minutes. Then, tissues were counterstained with hematoxylin and dehydrated through graded alcohols. Finally, the cover slip was mounted on the tissues with Permount after three successive xylenes baths.

Two digital pictures (×200) were randomly taken by Dr. Umphrey, a board-certified pathologist, in a blinded manner for each tumor slice that had undergone TUNEL, Ki67, ERα, or ERβ staining using SPOT camera on a Nikon Optiphot-2 microscope (Nikon inc., Melville, NY), interfaced with personal computer and SPOT software. The apoptotic (TUNEL) cells were segmented by color difference between the target cells (brown) and non-target cells (blue) or background (pale pink), while tumor cells (either apoptotic or non-apoptotic) were segmented by brightness and roundness differences, and then counted in all two pictures per tumor. Apoptotic cell density (%) was calculated by the ratio of the number of apoptotic cells to the total number of tumor cells. The proliferative (Ki67), ERα, and ERβ expressing cells were segmented by the signal-intensity difference, while the intensity threshold was manually determined; since the boundaries of the non-target cells were often vague, the cell densities were calculated by the number of target cells per unit area (N/mm^2^) instead. Uneven background intensity was corrected using “Rolling Ball” algorithm [20], while the radius was manually determined. The image segmentation and cell counting were implemented using ImageJ version 1.45i.

### Statistical analysis

One-way ANOVA [21] was carried out using SAS, version 9.2 (SAS Institute Inc., Cary, NC) to compare the apoptotic (TUNEL), proliferative (Ki-67), ERα, and ERβ cell densities among groups. Multivariate Pearson correlation coefficients were found using SAS version 9.2 (SAS Institute Inc., Cary, NC) to examine the correlation between the changes of ADC values and tumor volumes, and the correlation between the densities of cells with histologic markers and the changes of tumor volumes or ADC values [22]. Two way repeated-measure (RM) ANOVA [23] was carried out using SPSS version 16.0 (SPSS Inc., Chicago, IL) to compare the changes of intratumoral ADC values or tumor volumes among different sub-groups (tamoxifen sensitive, intermediate, and resistant groups) during the therapeutic period. *p* values less than 0.05 were considered significant. Data are presented as means±standard error. Dr. Beasley implemented all statistical analyses.

## Results

**Figure 2:**
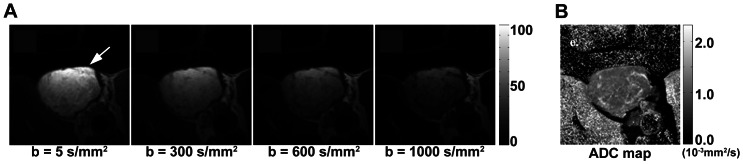
Diffusion weighted images and ADC map. (A) Representative diffusion-weighted images of an MNU-induced mammary tumor prior to the initiation of tamoxifen therapy with four *b* factors such as 5, 300, 600, and 1000 s/mm^2^, when the same gray scale was applied, and (B) ADC map calculated from those images.

Diffusion weighted images of MNU-induced mammary tumors were successfully obtained with minimal motion artifact. Figure 2 shows representative diffusion weighted images of a tumor prior to tamoxifen therapy initiation at four different *b* values of 5, 300, 600 and 1000 s/mm^2^, when the same gray scale was applied, and the ADC map of the segmented tumor region obtained from the four images. Tumor is indicated with a white arrow in Figure 2A.

**Figure 3:**
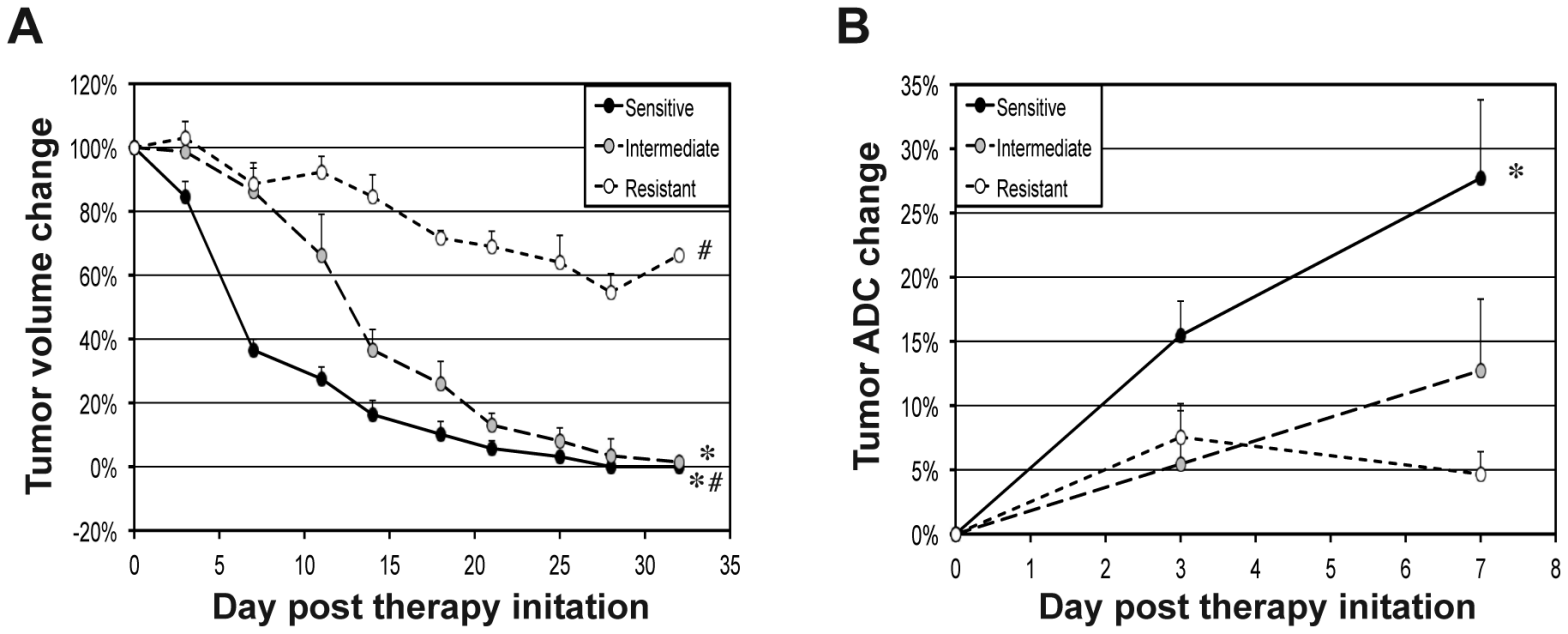
Tumor volume and ADC changes following tamoxifen therapy. (A) Volume changes of tumors sensitive, intermediate, or resistant to tamoxifen over 32 days after therapy initiation, when the initial tumor volumes were normalized to 100%. (B) ADC changes of sensitive, intermediate, or resistant tumors for 7 days after therapy initiation, when the initial ADC values were normalized to 0%. Statistical differences from resistant and intermediate groups are represented with asterisk and hash mark, respectively.

Differential therapy effect was induced by medium dose of tamoxifen and significantly correlated with early ADC changes of tumor region. Figure 3A shows the tumor-volume changes of the three sub-groups over the 4 weeks of therapy period of group 1, when the initial tumor volume (2.17±0.30 cm^3^) was normalized to 100%; the therapy durations for 50% volume reduction of sensitive, intermediate, and resistant tumors were 5.9±0.3 days (n = 5), 12.3±1.5 days (n = 6), and more than 28 days (n = 4), respectively. Figure 3B shows the ADC changes of the three sub-groups for 7 days, when the initial ADC values (9.48±0.18×10^−4^ mm^2^/s) were normalized to 0%. The mean ADC changes of sensitive, intermediate, and resistant tumors for 3 days after therapy initiation were 15.5±2.7%, 5.5±4.2%, and 7.5±2.6%, respectively, while those for 7 days were 27.7±6.1%, 12.7±5.6%, and 4.7±1.8%, respectively. Asterisk and hash mark represent statistical difference from the resistant and intermediate groups, respectively. Table 1 summarizes the correlation between the early change of tumor ADC value (either 3 or 7 days) and the tumor-volume change for a longer term; the ADC change for 3 days (ADC_3D_) was significantly correlated with the tumor-volume change until day 11, but the significant correlation between the ADC change for 7 days (ADC_7D_) and the tumor-volume change was observed until day 18.

**Table 1:** *p* values presenting the statistical correlation between tumor volume and ADC changes during 3∼32 days after therapy initiation (*r* values are inserted in parenthesis).

	Tumor volume change								
	3 days	7 days	11 days	14 days	18 days	21 days	25 days	28 days	32 days
ADC change (3 days)	**0.0139** (−0.66)	**0.0064** (−0.71)	**0.0083** (−0.70)	0.1890 (−0.39)	0.6177 (−0.16)	0.8502 (0.06)	0.6633 (0.14)	0.7906 (−0.09)	0.9725 (−0.01)
ADC change (7 days)		**0.0010** (−0.80)	**0.0003** (−0.85)	**0.0052** (−0.72)	**0.0170** (−0.67)	0.0795 (−0.53)	0.1131 (−0.48)	0.1598 (−0.45)	0.2303 (−0.39)

The significant correlation between the changes of ADC value and tumor volume was observed for group 2 as well. For group 2, the tumor volumes of sensitive and intermediate sub-groups decreased 61.5±4.5% (n = 4) and 27.0±6.2% (n = 3), respectively, but that of resistant sub-group increased 24.4±16.8% (n = 3), during 7 days of therapy; statistical significance was detected between sensitive and resistant groups (p = 0.0138), but not among the others. ADC_3D_ of sensitive, intermediate, and resistant tumors were 11.6±3.2%, 2.4±6.0%, and −3.4±0.3%, while ADC_7D_ of those groups were 26.7±7.8%, 9.5±5.5%, and −8.0±2.6%, respectively; similar to the tumor-volume change, statistical significance was detected only between sensitive and resistant groups (p = 0.0058). Also, ADC_3D_ was significantly correlated with the tumor-volume change for either 3 or 7 days (p = 0.0190; r = −0.79 and p = 0.0176; r = −0.80, respectively), but the higher correlation was observed between ADC_7D_ and tumor-volume change for 7 days (p = 0.0032; r = −0.89). The initial mean tumor volume and ADC value of group 2 were 4.16±0.49 cm^3^ and 9.34±0.18×10^−4^ mm^2^/s, respectively.

**Figure 4:**
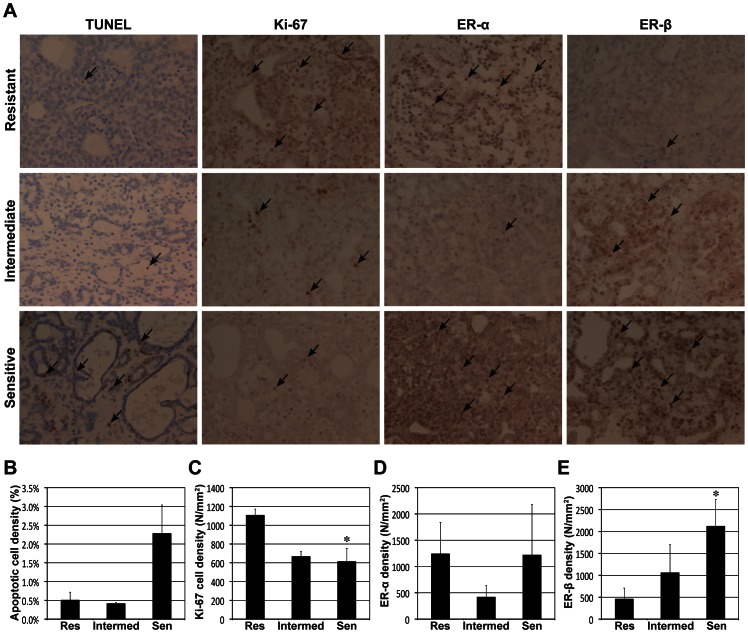
Histological analysis of tumor response following tamoxifen therapy. (A) Representative microphotographs of tamoxifen sensitive, intermediate, and resistant tumor tissues with TUNEL, Ki-67, ERα, and ERβ staining, while the target cells were indicated with black arrows. (B) Apoptotic (TUNEL), (C) proliferative (Ki-67), (D) ERα-positive, and (E) ERβ-positive cell densities of the three groups. Statistical difference from resistant group is represented with asterisks above bars.

**Figure 5:**
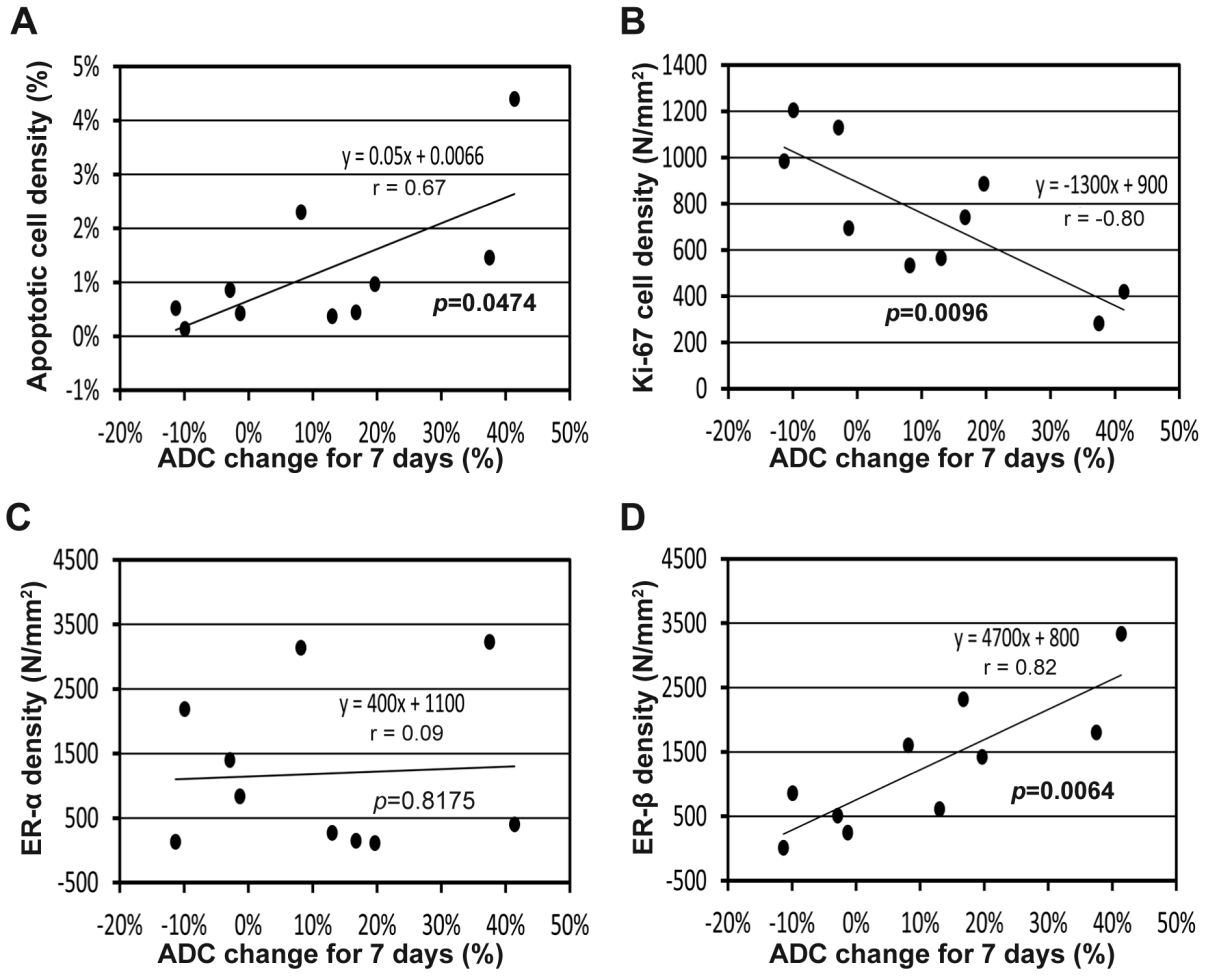
Correlation between ADC change for 7 days (ADC_7D_) and histological findings. (A) ADC_7D_ vs. apoptotic (TUNEL) cell density (%). (B) ADC_7D_ vs. proliferative (Ki-67) cell density (N/mm^2^). (C) ADC_7D_ vs. ERα-positive cell density (N/mm^2^). (D) ADC_7D_ vs. ERβ-positive cell density (N/mm^2^).

The early ADC change was significantly correlated with apoptotic, proliferating, and ERβ-positive cell densities, but not with ERα-positive cell density. Figure 4A shows representative photomicrographs of tumor tissues with TUNEL, Ki-67, ERα, and ERβ staining in each sub-group of group 2, and the target cells were indicated with black arrows in each sub-figure. Figures 4B to 4E present the mean apoptotic (TUNEL), proliferative (Ki-67), ERα-positive, and ERβ-positive cell densities, respectively, of the three sub-groups, while asterisks above the bars represent the statistical differences from resistant group; although no significant difference was detected in apoptotic and ERα-positive cell densities among groups, there was significant difference in proliferating and ERβ-positive cell densities between sensitive and resistant groups (p = 0.0164 and p = 0.0364, respectively). Figure 5 shows the correlation between ADC_7D_ and the cell densities; ADC_7D_ was significant correlated with apoptotic, proliferating, and ERβ-positive cell densities (p = 0.0474; r = 0.67, p = 0.0096; r = −0.80, and p = 0.0064; r = 0.82, respectively), but not with ERα-positive cell density (p = 0.8175; r = 0.09). The tumor-volume change for 7 days was significantly correlated with proliferating and ERβ-positive cell densities (p = 0.0042; r = 0.84 and p = 0.0390; r = −0.69, respectively), but not with apoptotic and ERα-positive cell densities (p>0.05). No correlation was observed between ADC_3D_ and any of the cell densities (p>0.05). The tumor-volume change for 3 days was significantly correlated with only ERβ-positive cell density (p = 0.0256; r = −0.73), but not with the others (p>0.05).

## Discussion

To our knowledge, this is the first report of DWI application for an MNU-induced breast-cancer murine model. To date, DWI studies of tumors located in mammary fat pad have achieved little success due to severe motion artifact. In this study, however, a simple method to separate a tumor from the chest motion was introduced, enabling to repeatedly obtain DW images with minimal motion artifacts even at high *b* values, and it can be also applied for orthotopic breast-cancer xenograft murine models easily. The plastic bars with wedges, however, may create pressure on the tumor region contacting the wedges altering tumor architecture and water diffusion. But, in this study, the plastic bars were applied parallel to the animal body as shown in Figure 1, and the length of wedge was only about 2 mm. The mean tumor size was about 20 mm in diameter, and only 7 axial image planes at the central region was selected for DWI. Therefore, the influence of wedges would be minimal to the mean ADC value of the central tumor region. There was a wide range of variation of tumor growth rate in this model, and ulceration was observed when tumors were large during long-term therapy monitoring. So, smaller size tumors were selected for long-term therapy group (group 1), relative to those of short-term therapy group (group 2). However, the initial intratumoral ADC values were about the same between groups 1 and 2.

The mechanism of apoptosis induced by tamoxifen has been well studied [24], [25], [26]. During the early phase of apoptosis, apoptotic volume decrease (AVD) occurs, and thereby water molecules within cell membrane are forced to be transferred out; since the water molecules in extra-cellular space have higher mobility, the water diffusion increases accordingly, which can be measured using DWI. The early ADC increase can be used as a prognostic indicator of tamoxifen therapy, because it was linearly proportional to the magnitude of treatment effect assessed by tumor-volume regression. In this study, either ADC_3D_ or ADC_7D_ was significantly correlated with long-term tumor volume change, but ADC_7D_ presented higher accuracy presumably because the extra-cellular water molecules were gradually increased during 7 days of therapy for sensitive tumors.

However, care should be taken to propose 7 days post therapy initiation as the optimal DWI time point to evaluate tamoxifen efficacy, because ADC value is dependent not only on the amount of extra-cellular water molecules, but also on interstitial fluid pressure (IFP); the higher IFP a tumor has, the more rapidly water molecules can be pushed out of the ROI. Tumor IFP is mainly determined by microvasculature [27], [28]. Since breast tumors present a wide range of vascularity [29], the intratumoral ADC increase may need to be interpreted accordingly for more accurate prognosis. In fact, both tumor IFP and vascularity can be measured based on dynamic contrast-enhanced MRI (DCE-MRI) [30], [31]. The relationship between ADC value and tumor IFP may need to be further investigated using combined modality with DCE-MRI and DWI to readjust the optimal DWI time point in both preclinical and clinical settings.

MNU-induced mammary tumors expressed both ERα and ERβ, and the therapeutic efficacy was associated with the level of ERβ, not ERα. However, one study showed that ERβ level assessed by immunohistochemistry was significantly correlated with the improved survival of ERα-negative breast cancer patients during 2-years tamoxifen adjuvant therapy, but not with that of ERα-positive breast cancer patients [32]. This discrepancy might be explained by the results of a recent study by Razandi *et al* [33]; tamoxifen targeted ERβ to induce apoptosis via increasing reactive oxygen species (ROS) in breast-cancer cell lines sensitive to tamoxifen therapy, but it functioned as an agonist to ERβ in resistant cell lines augmenting cell proliferation. The transition mechanism of ERβ role according to tamoxifen sensitivity has not been understood yet, but, anyhow, ERβ may not be suitable for a primary prognostic biomarker to predict tamoxifen therapy efficacy for random patients. However, the level of ERβ might be considered as a secondary prognostic biomarker to validate the therapeutic efficacy assessed by DWI.

## Conclusion

The feasibility of DWI for early evaluation of tamoxifen therapy was demonstrated for ER-positive breast cancer using a human-like rat model; identification of tamoxifen-sensitive patients using DWI may enable personalized tamoxifen treatment. The significant correlation between early ADC change and ERβ-positive cell density suggests that ERβ may play an important role as a therapeutic indicator of tamoxifen.
